# Genetic Diversity and Conservation Status of *Helianthus verticillatus*, an Endangered Sunflower of the Southern United States

**DOI:** 10.3389/fgene.2020.00410

**Published:** 2020-05-15

**Authors:** Tyler P. Edwards, Robert N. Trigiano, Bonnie H. Ownley, Alan S. Windham, Christopher R. Wyman, Phillip A. Wadl, Denita Hadziabdic

**Affiliations:** ^1^Department of Entomology and Plant Pathology, The University of Tennessee, Knoxville, Knoxville, TN, United States; ^2^Department of Plant Sciences, The University of Tennessee, Knoxville, Knoxville, TN, United States; ^3^United States Department of Agriculture, Agriculture Research Service, U.S. Vegetable Laboratory, Charleston, SC, United States

**Keywords:** bottleneck, diversity, conservation, genetic diversity, microsatellite loci, multilocus genotypes

## Abstract

Evaluating species diversity and patterns of population genetic variation is an essential aspect of conservation biology to determine appropriate management strategies and preserve the biodiversity of native plants. Habitat fragmentation and potential habitat loss are often an outcome of a reduction in naturally occurring wildfires and controlled prescribed burning, as seen in *Helianthus verticillatus* (whorled sunflower). This endangered, wild relative of the common sunflower, *Helianthus annuus*, is endemic to four locations in Alabama, Georgia, and Tennessee, United States. Despite its endangered status, there is no recovery plan for *H. verticillatus*, and knowledge related to its basic plant biology and importance in ecosystem services is mostly unknown. In this study, we utilized 14 microsatellite loci to investigate fine-scale population structure and genetic diversity of *H. verticillatus* individuals found on two sampling sites within the Georgia population. Our results indicated moderate genetic diversity and the presence of two distinct genetic clusters. Analyses of molecular variance indicated that the majority of variance was individually based, thus confirming high genetic differentiation and limited gene flow between *H. verticillatus* collection sites. The evidence of a population bottleneck in these sites suggests a recent reduction in population size that could be explained by habitat loss and population fragmentation. Also, high levels of linkage disequilibrium were detected, putatively suggesting clonal reproduction among these individuals. Our study provides a better understanding of fine-scale genetic diversity and spatial distribution of *H. verticillatus* populations in Georgia. Our results can underpin an original recovery plan for *H. verticillatus* that could be utilized for the conservation of this endangered species and to promote its persistence in the wild.

## Introduction

*Helianthus verticillatus* (Small), commonly known as the whorled sunflower, is a herbaceous perennial endemic to four locations in the southeast United States (Matthews et al., 2002; Chafin and Owers, 2010). Originally discovered in 1892 near the town of Henderson in Chester County, Tennessee, United States (Seiler and Gulya, 2004), the plant was initially labeled as *Helianthus schweinitzii*, and later annotated as *H. verticillatus* by Small (1898) (Matthews et al., 2002). After the original discovery, the plant was not observed in the wild again until 1994, where it was identified near the Coosa River in Floyd County, Georgia, United States (Matthews et al., 2002). Soon after, two more populations were discovered, the first in Cherokee County, Alabama, United States in 1996, and the latter in Madison County, Tennessee, United States in 1998 (Matthews et al., 2002; Ellis et al., 2008).

*Helianthus verticillatus* is morphologically similar to three other *Helianthus* spp., *Helianthus angustifolius* (narrow-leaved or swamp sunflower), *Helianthus divaricatus* (woodland sunflower), and *Helianthus microcephalus* (small-headed sunflower), with shared traits of rounded hairy leaves and small flower heads (Chafin and Owers, 2010). In contrast to the three aforementioned species, *H. verticillatus* usually has three-to-four leaves, which are arranged in a whorled, opposite pattern (Matthews et al., 2002; Chafin and Owers, 2010). *H. verticillatus* preferentially grows in prairie-like habitats, open flood plains, and wet depressions near the edges of forests (Chafin and Owers, 2010) in large clonal clumps that can reach 3 m in height (Matthews et al., 2002; Chafin and Owers, 2010). The vigorous growth and showy yellow flowers of *H. verticillatus* may render the species a potential valuable ornamental plant and presumably a useful pollinizer in the wild as well as in the home garden, which is common for other *Helianthus* spp. (Schmidt et al., 1995; Chafin and Owers, 2010).

This species was first speculated to be a hybrid of two other *Helianthus* species, *Helianthus eggertii* and *H. angustifolius* (Beatley, 1963; Matthews et al., 2002). However, this theory was discarded due to the difference in chromosome number between *H. eggertii* (*n* = 51) and *H. angustifolius* (*n* = 17) (Matthews et al., 2002). Due to similar morphology and overlapping habitats among *H. verticillatus* and other *Helianthus* spp., Heiser et al. (1969) speculated that *H. verticillatus* was a hybrid between *H. angustifolius* and *H. grosseserratus*. Both species belong in the *Atrorubens* section of the genus, have similar chromosome number (*n* = 17), overlapping habitats, and *H. grosseserratus* displayed verticillate leaf patterns in “exceptions” (Beatley, 1963; Heiser et al., 1969; Matthews et al., 2002; Seiler and Gulya, 2004; Ellis and McCauley, 2009).

To test the Heiser et al. (1969) hybrid theory, genetic diversity and population structure of *H. verticillatus* were investigated using nuclear and chloroplast DNA microsatellite loci (Ellis et al., 2006). Their results indicated moderate genetic diversity of *H. verticillatus* populations from three locations in Alabama, Georgia, and Tennessee (Ellis et al., 2006). In addition, they observed significant differences between populations of *H. verticillatus* and the proposed ascendant species (Ellis et al., 2006). *Helianthus verticillatus* could be a hybrid due to frequent hybridization within the genus, but did not descend from the specific cross between *H. grosseserratus* and *H. angustifolius* (Rieseberg, 1991; Ellis et al., 2006). Ellis et al. (2006) rejected the hypothesis that *H. verticillatus* was a hybrid, thus proposing that the plant was a distinct species. Further research focusing solely on this plant found high clonal diversity, despite predominate vegetative reproduction (Mandel, 2010). Moreover, there were far fewer distinct genetic individuals than previously thought (Ellis et al., 2006; Mandel, 2010). Additional studies on *H. verticillatus* have indicated very apparent differences in fitness among populations (Mandel, 2010; Ellis et al., 2006, 2008; Ellis and McCauley, 2009). These studies also posit that *H. verticillatus* may be more prone to self-pollination and may have experienced a major decline in distinct genetic individuals in recent history (Mandel, 2010).

As a result of these studies and documented habitat loss due to agricultural expansion and timber harvest (USFWS, 2014), in 2014 *H. verticillatus* was classified as a federally endangered species. There are currently only four known populations of *H. verticillatus*, two in Tennessee (TN), McNairy and Madison Counties, and two near the Georgia (GA)-Alabama (AL) border (Floyd County, GA and Cherokee County, AL), roughly 3.5 km apart (Ellis, 2008; Mandel, 2010). In Madison County, TN, the *H. verticillatus* population can be sub-divided into two subpopulations less than 1.5 km apart; one very dense subpopulation located near TN Highway 45 and the other a sparse subpopulation located near railroad tracks and agricultural installations. The McNairy County, TN population is about 50 km south of Madison County and can be divided into two distinct populations along Prairie Branch Creek (Ellis et al., 2008). The AL and GA populations are located on timberland owned by Weyerhaeuser Corporation. The habitats of the AL and GA populations differ greatly from the TN populations because they are wet prairies on undeveloped, scantily populated land (Ellis et al., 2008; Ellis and McCauley, 2009; Mandel, 2010). In addition, these *H. verticillatus* populations are currently managed and protected by The Nature Conservancy. The current management plan for these populations includes controlled burns on land inhabited by *H. verticillatus* and frequent checks on population fitness (Malcolm Hodges, The Nature Conservancy, personal communication).

Although some progress has been made investigating the genetic diversity in *H. verticillatus* populations, our knowledge concerning the basic biology, census data, and a lack of a well-defined conservation plan remain a major problem for preservation of this species (Ellis et al., 2006; Ellis, 2008). The United States Fish and Wildlife Service (USFWS) with the United States Department of the Interior (USDOI) have designated the land on which this sunflower is endemic as a critical habitat (USFWS, 2014). This designation provides only limited support for these plants because, as outlined by the Endangered Species Act (ESA), critical habitats have little effect on the land if federal funds are not involved (ESA, 1973). Because of this, corporate and private landowners (i.e., The Nature Conservancy and the Weyerhaeuser Corporation in the case of the AL and GA populations) have complete control over protection of this plant, including but not limited to provision of funds for habitat maintenance as well as best management practices to landowners for this species. However, there is currently no funding to protect the TN *H. verticillatus* populations, leaving these plants completely vulnerable to extinction.

Understanding the current state of spatial dynamics and genetic diversity of *H. verticillatus*, coupled with experimental data of historical genetics, could prove to be paramount for conservation of this species, as it has with other species in similar predicaments (Brzosko et al., 2002; Walck et al., 2002; Willi et al., 2006; Bowen, 2011). Ecosystem services depend on several factors including a number of unique life forms, their equitability, genetic variability, biodiversity, and potential extension risk (Cardinale et al., 2012). By protecting the diversity of the ecosystems in which this species inhabit, we maintain its equilibrium and therefore, the ability of the ecosystem to function (Cardinale et al., 2012). To better understand how to enhance conservation efforts for *H. verticillatus*, this study utilized microsatellite loci to determine genetic variation and spatial dynamics of the few remaining small populations of this plant while trying to discern the effect of high levels of clonality within them (Pashley et al., 2006).

In this study, we focused on fine-scale population structure and genetic diversity of *H. verticillatus* growing on privately owned land. In studies performed on a large geographic scale, although populations were isolated and reproduced mainly clonal via rhizomes, there was high genetic diversity within the populations (Ellis et al., 2006; Ellis and McCauley, 2009; Mandel, 2010). These studies also found high clonal diversity and the presence of polymorphic genotypes demonstrating predominantly clonal reproduction (Ellstrand and Roose, 1987; Mandel, 2010). Focusing on this plant at a smaller geographical scale could give new insights into the recent history, mating systems, and further refine existing conservation plans for *H. verticillatus* (Ellis, 2008).

The primary objective of this study was to assess clonal diversity and spatial structure of *H. verticillatus* of two geographically close populations in GA. Based on the biology and previous research on *H. verticillatus*, our hypothesis was that there were diminished numbers of distinct genets within the sites sampled. However, we also hypothesized that these two sites will harbor plants with high genetic diversity and should be spatially structured into discrete genetic clusters. With expanded knowledge of the fine-scale genetic diversity and population structure of these two populations of *H. verticillatus*, augmenting current plans and the creation of a solid recovery plan will be much more feasible. Successful plant conservation efforts including, but not limited to *Potentilla robbinsiana* (Robbins’ Cinquefoil) and *Echinacea tennesseensis* (Tennessee Purple Coneflower) (USFWS, 2002) could be used as model systems for a recovery plan and preservation of biodiversity in *H. verticillatus* fragmented populations. The plan could include not only the identification of specific populations that could be used in breeding programs, but outreach and educational efforts, improved *in vitro* propagation methods, transplanting into native habitats and botanical gardens, and potential commercialization of *H. verticillatus*.

## Materials and Methods

### Sample Collection

*Helianthus verticillatus* leaf samples (*n* = 206) were collected from two sites located near Cave Spring, GA, which is managed and maintained by The Nature Conservancy. Within this location, there are only two discrete areas where endangered *H. verticillatus* was growing. We collected samples from both sites. Site one consisted of five contiguous 1 × 1 m quadrants of *H. verticillatus*. All stems were counted in each quadrant and five leaves per stem were collected at random from no more than 30% of the individual stems. Site one yielded 74 samples, 27 from the first quadrant, 17 from the second, 5 from the third, 11 from the fourth, and 14 from the fifth quadrant (Supplementary Figure S1). Site two was non-contiguous, less than 1 km southeast of site one in a meadow, surrounded by pine plantation, and encompassing an area about 60 × 30 m. This plot was divided into a 3 × 3 m grid (Supplementary Figure S1) with five leaf samples taken from one individual stem at the intersects of each quadrant and from one individual stem at the center of the quadrant (Escaravage et al., 1998; Supplementary Figure S1). This sampling method was adapted from Escaravage et al. (1998) due to the clonal nature of this plant to only sample a single genet at each intersect. Some intersects did not contain *H. verticillatus* specimens and were excluded from this study. The second site yielded a total of 132 samples.

The sampling methods used at both sites were different for multiple reasons including spatial structure of these populations. With the forested barrier, the individual sampling sites, in theory, should cluster together. At the first site, the 1 × 1 m quadrants were contiguous to assess the potential clonal spread via rhizomes observed in previous studies (Lienert, 2004; Ellis et al., 2006, 2008; Ellis, 2008; Ellis and McCauley, 2009; Mandel, 2010). At site two, the sampling locales (grid intersects) were separated by 3 m with exception of the center-grid (∼1.5 m from the intersections) locations. This method was employed for two main reasons: (1) a dense population of *H. verticillatus* has never been sampled this rigorously in previous studies and (2) to avoid collecting genetically identical individuals, per previous studies, and remove potential bias caused by clonality from this portion of the study (Lienert, 2004; Ellis et al., 2006, 2008; Ellis, 2008; Ellis and McCauley, 2009; Mandel, 2010). In addition, these were the only two populations managed by The Nature Conservancy at this location. All samples (*n* = 206) were placed in plastic bags containing silica gel at a ∼10:1 ratio (silica gel to plant material weight) and stored on ice at the time of sampling to prevent degradation (Chase and Hills, 1991). These samples were then stored at −80°C until DNA extractions were completed.

### DNA Extraction

Leaf tissue from each sample was placed into sterile 2-ml conical screw-cap microcentrifuge tubes (Fisherbrand, Pittsburgh, PA, United States) with sterile 13-mm zirconia/silica beads (BioSpec Products, OK, United States), and submerged into liquid nitrogen for 2 min. Samples were homogenized using a Bead Mill 24 (Thermo Fisher Scientific, Walther, MA, United States) for 20 s twice with 5 min in liquid nitrogen between the homogenization steps. DNA extraction was completed using a modified protocol of the DNeasy Plant Mini kit (Qiagen, Valencia, CA, United States) by including 2% v/v liquid polyvinylpyrrolidone and 4 μl of RNase A in the lysis buffer. After addition of P3 buffer, samples were frozen (−20°C) for 1 h. Genomic DNA was quantified using a NanoDrop ND-1000 spectrophotometer (NanoDrop Technologies, Wilmington, DE, United States) and stored at −20°C.

### Selection of Microsatellite Loci and Polymerase Chain Reaction

Forty eight microsatellite loci developed from *H. annuus* (Ellis et al., 2006; Pashley et al., 2006; Ellis, 2008; Mandel, 2010) were screened for the presence of polymorphisms, amplification, and consistency among the collected samples of *H. verticillatus.* From that initial screen, fifteen tri- and tetra-repeat microsatellites were selected for this study. One locus, HV022, was later removed because it was uninformative. Therefore, a total of 14 loci were used to evaluate genetic diversity of *H. verticillatus.* Polymerase chain reaction (PCR) was completed in 10 μl reactions with 1 μl of (10 ng/μl) genomic DNA, 2.5 μl (10 μM) of both forward and reverse primers, 0.5 dimethyl sulfoxide (Thermo Fisher Scientific, Waltham, MA, United States), 4 μl (0.8×) GoTaq Colorless Master mix (Promega, Madison, WI, United States), and brought to a final volume of 10 μl with sterile Nanopure water (Thermo Fisher Scientific). Reactions were performed using a Mastercycler Pro Automatic Thermal Cycler (Eppendorf Biotech Co., Hamburg, Germany) using the following touchdown-PCR (TD-PCR) conditions: 95°C for 3 min, followed by 10 cycles of 94°C for 30 s, 65°C lowering 1°C per cycle to a final 55°C for 30 s, then 72°C for 45 s, another 30 cycles of 94°C for 30 s, 55°C for 30 s, 72°C for 45 s and a final elongation step at 72°C for 20 min (Ellis, 2008; Korbie and Mattick, 2008). Amplicons were analyzed on a QIAxcel Capillary Electrophoresis system (Qiagen, Valencia, CA, United States) using a 15–600 base pair (bp) internal marker and scored with a 25 bp DNA size marker to assess raw allele length (Wang et al., 2009; Dean et al., 2013). A sample that amplified consistently with all primers, a positive control, was included in every 96-well plate as well as a negative control of sterile Nanopure water (Thermo Fisher Scientific) to check for consistency of results. If either the positive control failed to amplify, or the negative control amplified in any of the plates, the whole set of reactions was repeated.

### Population Diversity

FLEXIBIN v2 (Amos et al., 2007) was used to bin raw allelic data into allelic classes. The resulting dataset was used for all further analyses. Samples in this study (*n* = 206) were then grouped into 14 collection zones: 5 representing the 1 × 1 m quadrants sampled at the first site and 9 for each row of the grid at the second site (Supplementary Figure S1). All binned data were clone corrected using POPPR v2.1.1 (Kamvar et al., 2014) to remove any identical multilocus genotypes (MLGs) from each collection zone. Clone correction was used to avoid any biases that could be caused by identical MLGs in further analyses.

All subsequent data analyses were completed using the multiple packages in R (RStudio, 2012; R Core Team, 2016). The package POPPR v2.1.1 was used to calculate various genetic diversity indices including the Shannon-Weiner index of MLG diversity (H) and the index of association (Ia), which takes into account both allelic richness and evenness (E_5_) of a collection zone (Hill, 1973; Shannon, 2001). In addition, POPPR was used to calculate the standard index of association (${\bar{\text{r}}}_{\text{d}}$), a measure of linkage disequilibrium that is commonly used for estimating the amount of clonal reproduction (Agapow and Burt, 2001; De Meeûs and Balloux, 2004). For this analysis, we used 10,000 permutations for each collection zone. The number of private alleles, alleles found only in one collection zone (Szpiech and Rosenberg, 2011), and Nei’s genotypic diversity (H_exp_) (Nei, 1978) were also analyzed with POPPR. The package hierfstat v0.04-22 (Goudet, 2005) was used to calculate the pairwise population differentiation and Nei’s pairwise genetic distance was calculated with the package adegenet v2.1.1 (Jombart, 2008).

### Population Structure

Clustering and population structure of *H. verticillatus* were assessed with STRUCTURE v2.3.4 (Pritchard et al., 2000) using a Bayesian Markov Chain Monte Carlo (MCMC) method. The parameters used in STRUCTURE included a burn-in period of 500,000 with 500,000 MCMC repetitions of 30 iterations at *K* = 1−10. STRUCTURE HARVESTER web v0.6.94 (Earl and vonHoldt, 2012) was used to infer the optimum *K* value, using Evanno’s method (Evanno et al., 2005) to represent the most probable number of genetic clusters. POPHELPER web v1.0.10 (Francis, 2017) was utilized to visualize the optimum value of *K* from the previous analyses. BAPS v5.0 (Corander et al., 2008) was used to infer overall population structure of the sample set. This program allows users to specify the number of genetic clusters to be tested, depending on the hypotheses and research question(s) of interest (Corander et al., 2008). BAPS was used to test for the presence of two clusters among *H. verticillatus* individuals under the assumption that both collection sites would group into two distinct populations due to high levels of clonal propagation (Ellis et al., 2006; Ellis, 2008; Ellis and McCauley, 2009; Mandel, 2010). The R package PopGenReport v3.0.0 (Adamack and Gruber, 2014) was used to visualize the presence of genetic clusters test using the discriminant analysis of principal components (DAPC) (Jombart et al., 2010).

Genetic differentiation was calculated using an analysis of molecular variance (AMOVA) with Arlequin v3.5.2.2 (Excoffier and Lischer, 2010). To assess genetic differentiation of *H. verticillatus* samples in this study, the following four variance partitions were used: one in which all collection zones were analyzed as a single hierarchical group, another where the collection zones were analyzed by collection site, one which was analyzed with STRUCTURE results, and a final one analyzed according to DAPC assignment. Bruvo’s distance, which estimates genetic distance between individuals rather than between collection zones (Bruvo et al., 2004), was calculated using POPPR and subsequently applied to create a minimum spanning network (MSN).

### Demographic History

The program BOTTLENECK v1.2.02 (Cornuet and Luikart, 1996) was used to determine whether or not there had been a recent bottleneck or expansion among *H. verticillatus* collection zones. Sign and Wilcoxon tests were employed to establish whether or not the loci used in this study were in the mutation-drift equilibrium (Cornuet and Luikart, 1996). The Sign test posits a null hypothesis of differences between observed and expected heterozygosity, whereas the Wilcoxon test assumes a null hypothesis of no significant excess of heterozygosity (Luikart et al., 1998; Piry et al., 1999). The following three mutation models were used with 10,000 iterations each: infinite allele model (IAA), stepwise mutation model (SMM), and the two-phase model (TPM) at default settings (Piry et al., 1999). The data was analyzed grouping samples by site as well as according to STRUCTURE and DAPC results.

## Results

### Determining Polymorphic Loci

Fourteen tri- and tetra-repeat microsatellites of the 48 primer pairs evaluated were polymorphic and amplified DNA in all tested samples (Table 1). The remaining loci were not included based on weak or no reactions, allele sizes not in predicted ranges, or the lack of polymorphisms. The 14 microsatellite loci were selected for further population assessments and binned prior to data analyses. Binning the lengths of the amplicons produced 2 to 16 different allelic classes for each of the 14 loci (Table 1). The amplicon size difference of these primers varied greatly over these classes, ranging from 4 (HV41) to 66 (HV42) base pair differences.

**TABLE 1:** Fourteen microsatellite loci used to examine genetic diversity and spatial population structure of *Helianthus verticillatus*.

Core locus^A^	Renamed locus^B^	Forward and reverse primers 5′–3′	Repeat motif	Number of alleles	Allele size range (bp)
BL0006	HV006	F: CATGGGTGATCAATGGAGTG	(GTGA)_3_	14	225–279
		R: CGGCACATAACAAGTGCTTC			
BL0013	HV012	F: CGAGACGGTTAAGAGCTTGC	(GTTA)_3_	16	319–364
		R: GGTGTACAACCAACTCACACC			
BL0022	HV017	F: ACTTACCGTTGCATTTGGTG	(TAA)_4_	3	105–111
		R: GCTTATCCCTAGAACACGATTACAG			
BL0015	HV024	F: AATTGGAGCGGATGGTATTG	(ATG)_4_	5	356–369
		R: AATATCTCTTATTTCAATAGTCCAACG			
BL0019	HV026	F: GAGTCCTGGCCTGAACAGAG	(GAAA)_3_	8	292–316
		R: CAAACTGCAATGTACCTTCTTGAC			
BL0024	HV028	F: CTCCCGCACTTCAAGCTAAC	(GTAA)_3_	5	117–126
		R: CATACACCTTTGCGGTTTCC			
BL0031	HV031	F: CCGGAAGATAACGACGAGTG	(GAC)_4_	10	405–437
		R: TCCATCGCTTTCCCTAAATC			
BL0033	HV033	F: GGGAGTTACACGCCTCCAG	(CAC)_4_	5	270–284
		R: CACAACCATACGCCATCAAG			
BL0034	HV034	F: GGTCGTCTACTACGGCTTCG	(TGTT)_4_	4	155–165
		R: TAACCGAACGACCATTCTTC			
BL0037	HV037	F: GGTTAGGGTGAGGGTGGTG	(TGCA)_3_	7	153–179
		R: AAGCCATAGTAAGTTCCTCTTACAAAC			
BL0041	HV041	F: ACATTTGGACGTTTGGAAGC	(CTT)_4_	2	185–189
		R: TCCATCGAGATGTTGACACG			
BL0042	HV042	F: GGTTACAACGGTGGAAGTCG	(GGC)_4_	16	364–430
		R: TCCGGTTCACCAATTCATTC			
BL0046	HV046	F: GAACCAACACAACCAAATCC	(AACA)_3_	10	312–339
		R: TGTCGCTTCAACGCATAAAC			
BL0048	HV048	F: TTGTGGAGACGGTGAATGAG	(GAA)_4_	5	215–233
		R: TAACCGAACGACCATTCTTC			

*^*A*^All core loci taken from Pashley et al. (2006). ^*B*^Loci renamed for the purpose of this study only.*

### Population Diversity

Clone correction of the original dataset (*n* = 206) removed three samples of the same MLG from analyses. Samples removed through clone correction were from three collection zones (C1, C5, and R7). The clone corrected data (*n* = 203) was used for all subsequent analyses with 14 polymorphic loci. *H. verticillatus* individuals were divided into 14 different collection zones based on location and quadrant in which samples were gathered (C1–5 corresponded to the first collection site where five different quadrants were sampled; R1–9 corresponded to site two and the nine different rows from the sampling grid).

The Shannon-Weiner index of MLG diversity (H) ranged from 1.61 in collection zone C3 to 3.26 in collection zone C1, with an overall average of 2.58 (Table 2). Evenness (E_5_) was 1 for all collection zones and 21 private alleles were identified in 8 of the collection zones (Table 2). Only one collection zone from site one (C2) had two private alleles. In site two, most of the private alleles were found in all but two of the collection zones (R6 and R7) with the most being found in collection zone R9 (*n* = 7) (Table 2). Nei’s gene diversity (H_exp_) ranged from 0.36 (collection zone C5) to 0.59 (collection zone R8) with an average of 0.50. However, site one had lower gene diversity (0.44) compared to site two (0.53) on average. The standardized index of association (${\bar{\text{r}}}_{\text{d}}$) had an average value of 0.13 with a maximum value of 0.28 (zone R7) for all collection zones except for zone R6 (*P* = 0.07) (Table 2). Values of ${\bar{\text{r}}}_{\text{d}}$ were significantly greater than zero (${\bar{\text{r}}}_{\text{d}}$ = 0 indicates linkage equilibrium), and supported the presence of linkage disequilibrium, suggesting putative clonal reproduction among *H. verticillatus* individuals.

**TABLE 2:** Genetic diversity indices of *Helianthus verticillatus* samples from two sampling sites (C and R populations) analyzed as 14 collection zones using 14 microsatellite loci.

Collection zone	*N*	MLG	H	Pa	H_exp_	${\bar{\text{r}}}_{\text{d}}$	*P-value* (${\bar{\text{r}}}_{\text{d}}$)
C1	27	26	3.26	0	0.44	0.15	*P* < 0.001
C2	17	17	2.83	2	0.41	0.15	*P* < 0.001
C3	5	5	1.61	0	0.54	0.25	*P* < 0.001
C4	11	11	2.4	0	0.43	0.11	*P* < 0.001
C5	14	13	2.56	0	0.36	0.23	*P* < 0.001
R1	19	19	2.94	3	0.54	0.08	*P* < 0.001
R2	19	19	2.94	1	0.51	0.05	*P* < 0.001
R3	17	17	2.83	2	0.55	0.09	*P* < 0.001
R4	18	18	2.89	1	0.51	0.1	*P* < 0.001
R5	19	19	2.94	3	0.52	0.08	*P* < 0.001
R6	14	14	2.64	0	0.45	0.03	*P* = 0.07
R7	8	7	1.95	0	0.57	0.28	*P* < 0.001
R8	8	8	2.08	2	0.59	0.13	*P* < 0.001
R9	10	10	2.3	7	0.54	0.09	*P* < 0.001
**Total/Mean**	**206**	**203**	**2.58**	**21**	**0.50**	**0.13**	

**N*, total number of samples; MLG, number of multilocus genotypes observed after clone correction; H, Shannon-Wiener index of MLG diversity; Pa, number of private alleles in each population; H_*exp*_, Nei’s genotypic diversity corrected for sample size; r̄_*d*_, the standardized index of association.*

### Population Structure and Genetic Differentiation

**FIGURE 1:**
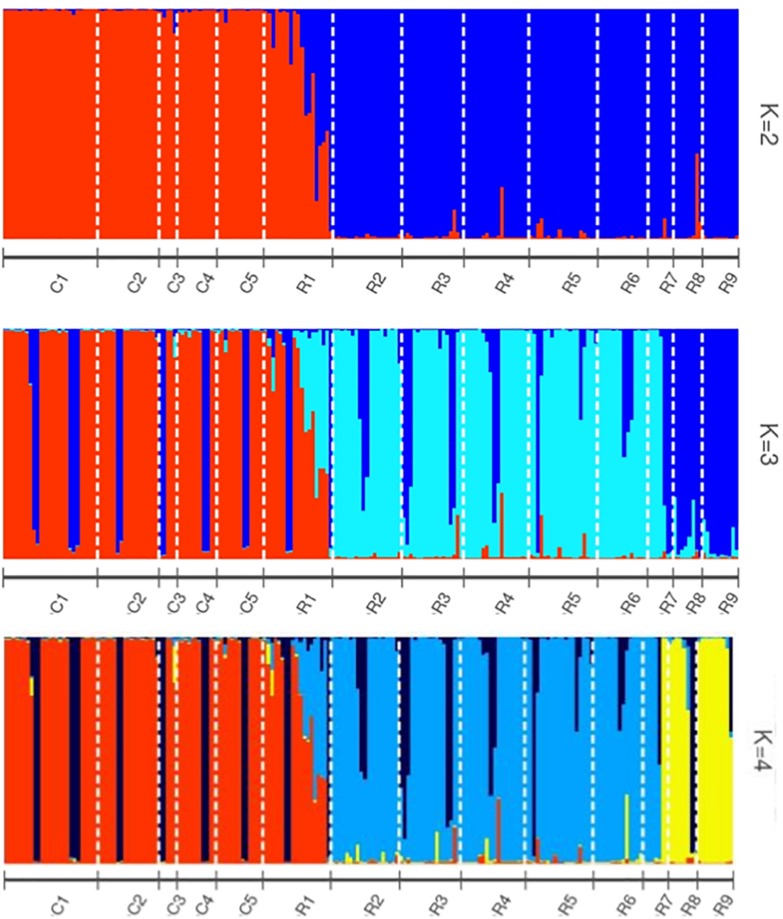
STRUCTURE bar graphs representing genetic clusters (*K* = 2–4) of samples from the two sampling sites (C and R) divided into 14 collection zones. Each bar represents an individual sample and colors code membership of each individual assigned cluster. Using Evanno’s method, the presence of two genetic clusters (*K* = 2) was found to be the best fit for this data.

*Helianthus verticillatus* individuals grouped into two distinct genetic clusters (Δ*K* = 2), based on the STRUCTURE results. STRUCTURE results were subsequently visualized using the POPHELPER v (Francis, 2017) platform (Figure 1). When the *H. verticillatus* samples were grouped into two clusters, (Δ*K* = 2), individuals from the R1 collection zone indicated the presence of admixture, which was very limited within other *H*. *verticillatus* collection zones (Figure 1). Though Evanno’s method indicated Δ*K* = 2 as the most probable results, data was also visualized as Δ*K* = 3 and Δ*K* = 4 (Figure 1). Clustering patterns for both indicated similar levels of admixture for the R1 collection zone, but additional admixture across other collection zones (Figure 1). BAPS produced similar results to STRUCTURE when analyzed as 2 clusters (*K* = 2) and 3 clusters (*K* = 3) (Supplementary Figure S2). However, these analyses did differ slightly in that BAPS R1 was clustered consistently with the C zones rather than its respective sampling site (Supplementary Figure S2).

The AMOVA of the collection zones as one hierarchal group indicated that most of the variation was found within the individuals (76.04%, *P* < 0.001; *F*st = 0.24) (Table 3). When data was analyzed with the collection zones as two distinct groups (C and R sampling sites), the majority of the variation was still found within individuals (50.96%, *P* < 0.001), rather than among individuals within collection zones (30.17%, *P* < 0.001), and among collection zones (18.87%, *P* < 0.001) (Table 3). When data was partitioned based on STRUCTURE results, the lowest variation was found among two collection zones (20.18%, *P* < 0.001; *F*st = 0.20), compared to among individuals within two genetic clusters (28.92%, *P* < 0.001), and within individuals (51%, *P* < 0.001) (Table 3). When samples were analyzed by grouping based on DAPC designation, the results were similar to those of the AMOVA when grouped by sampling site and STRUCTURE, with the majority of variation occurring within individuals (50.72%, *P* < 0.001) (Table 3).

**TABLE 3:** Analysis of molecular variance (AMOVA) for *Helianthus verticillatus* across 14 microsatellite loci for all the collection zones structured as one hierarchal group **(A)**, two groups separated by sampling site **(B)**, two clusters as indicated by STRUCTURE **(C)**, and two clusters as indicated by discriminate analysis of principal components (DAPC) results **(D)**.

Source of variation	d.f.	Sum of squares	Variance components	Percentage of variation	*P*-value
**A. Analysis of 14 collection zones as one hierarchal group**					
Among collection zones	13	449.39	1.08 Va	23.96	*P* < 0.001
Within collection zones	394	1351.7	3.43 Vb	76.04	*P* < 0.001
Total	407	1801.1	4.51		
Fixation indices: *F*st = 0.24					
**B. Analysis of 2 sampling sites (C and R sites)**					
Among collection sites	1	181.82	0.93 Va	18.87	*P* < 0.001
Among individuals within collection sites	202	1107.3	1.49 Vb	30.17	*P* < 0.001
Within individuals	204	512	2.50 Vc	50.96	*P* < 0.001
Total	407	1801.1	4.93		
Fixation indices: *F*st = 0.19, *F*is = 0.37, *F*it = 0.49					
**C. Analysis of 2 clusters as indicated by STRUCTURE results**					
Among clusters	1	206.02	0.99 Va	20.18	*P* < 0.001
Among individuals within clusters	202	1083.1	1.43 Vb	28.92	*P* < 0.001
Within individuals	204	512	2.51 Vc	50.99	*P* < 0.001
Total	407	1801.1	4.93		
Fixation indices: *F*st = 0.20, *F*is = 0.36, *F*it = 0.49					
**D. Analysis of 2 clusters as indicated by DAPC results**					
Among clusters	1	210.04	1.02 Va	20.67	*P* < 0.001
Among individuals within clusters	202	1079.05	1.42 Vb	28.61	*P* < 0.001
Within individuals	204	512	2.51 Vc	50.72	*P* < 0.001
Total	407	1801.09	4.95		
Fixation indices: *F*st = 0.21, *F*is = 0.36, *F*it = 0.49					

**F*st, variance among sampling sites relative to the total variance; *F*is, inbreeding coefficient of individuals relative to the population; *F*it, variance in the total population.*

**FIGURE 2:**
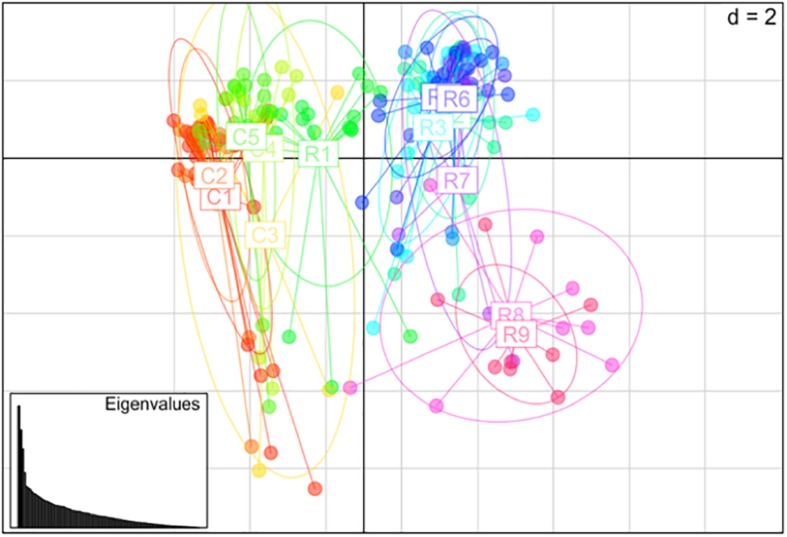
Principal coordinates analysis (PCoA) using discriminant analysis of principal components (DAPC) method among 14 collection zones (C1–5 collected from site 1 and R1–9 collected at site 2) of *Helianthus verticillatus* samples using 14 microsatellite loci.

**FIGURE 3:**
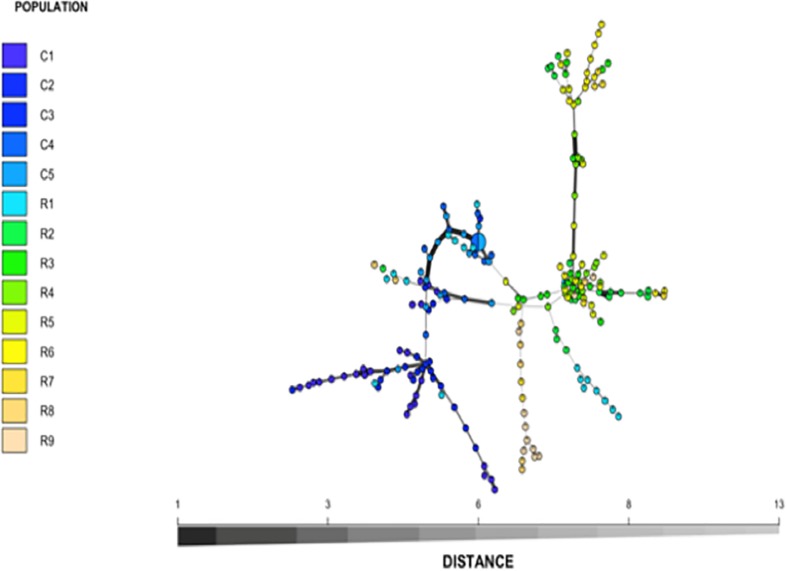
Minimum spanning network (MSN) of *Helianthus verticillatus* based on Bruvo’s genetic distance for 14 microsatellite loci. The nodes of the MSN represent individual multilocus genotypes (MLGs) with the size and color representing population membership size and designated collection zone, respectively. To avoid overlapping nodes, the size was scaled to log1.75*n*, where *n* equals the node sample size. Lines between nodes represent genetic distance between MLG as determined by Prim’s algorithm. Populations C1–5 were collected from site 1 while populations R1–9 were collected from site 2.

Nei’s pairwise genetic distance was lowest within the first sampling site, particularly between collection zones C4 and C5 (Supplementary Table S1). The highest genetic distance was found between collection zones C5 of the first sampling site and R9 of the second site (Supplementary Table S1). Pairwise population differentiation (*F*st) corresponded with Nei’s genetic distance, with the highest amount of differentiation between collection zones C5 and R9, and the lowest between collection zones C4 and C5 (Supplementary Table S2). DAPC findings matched the STRUCTURE and BAPS results clustering into two main groups, one predominately site one and site two with admixture at collection zone R1 (Figure 2). The MSN corresponded with BAPS, STRUCTURE and DAPC results, showing two distinct clusters with MLGs from zone R1 grouping closer to MLGs from the first sampling site (C1–5) (Figure 3). Genetic distance between MLGs in the MSN is represented by the thickness of the line between each MLG node (Kamvar et al., 2015). The size and color of each node represents the number of samples and collection zone representing each MLG, respectively (Kamvar et al., 2015).

### Demographic History

The program BOTTLENECK indicated the presence of a recent population bottleneck for all three grouping analyses (one group based on STRUCTURE results, one group based on collection zones and the last group based on the DAPC results). The Sign, Wilcoxon, and the standardized difference tests across three mutation models showed a significant heterozygote excess when the collection zones were grouped by sampling site, with both shifting from the mutation equilibrium model (Table 4). When the collection zones were grouped by STRUCTURE and DAPC assignments, the same results were observed (Table 4).

**TABLE 4:** Bottleneck determination by Sign tests for *Helianthus verticillatus* samples using 14 microsatellite loci and grouped by STRUCTURE results **(A)**, by sampling site **(B)** and by discriminate analysis of principal components (DAPC) results **(C)**.

**A. Bottleneck results when grouped by STRUCTURE results**					
	**Mutation model** **(excess/deficit)^A^**				
**STRUCTURE group**	**IAM**	**TPM**	**SMM**	**Mode-shift^B^**	***P*-value**

1	14/0	14/0	12/2	Shifted	*P* < 0.01
2	14/0	14/0	12/2	Shifted	*P* < 0.01
**B. Bottleneck results when grouped by collection zone**						
	**Mutation model** **(excess/deficit)^A^**				
**Collection zone**	**IAM**	**TPM**	**SMM**	**Mode-shift^B^**	***P*-value**

R	14/0	14/0	12/2	Shifted	*P* < 0.01
C	14/0	14/0	12/2	Shifted	*P* < 0.01
**C. Bottleneck results when grouped by DAPC results**					
	**Mutation model** **(excess/deficit)^A^**				
**DAPC group**	**IAM**	**TPM**	**SMM**	**Mode-shift^B^**	***P*-value**

1	14/0	14/0	12/2	Shifted	*P* < 0.01
2	14/0	14/0	12/2	Shifted	*P* < 0.01

*IAM, infinite allele model; TPM, two-phase mutation model; SMM, stepwise mutation model. ^*A*^Excess/deficit indicates the number of loci showing excess/deficit of gene diversity under mutation-drift equilibrium. ^*B*^A shift in the distribution of allelic frequency classes is expected in populations that experienced recent bottleneck event.*

## Discussion

### Population Structure, Clonality, and Genetic Diversity

Our results indicated moderate genetic diversity, high levels of clonality, the presence of spatial structure, and evidence of recent bottleneck among *H. verticillatus* individuals, thus supporting our hypothesis of clonality and population structure. Both sampling sites harbored moderate levels of genetic diversity and in contrast to our proposed hypothesis, a high number of distinct genets (*N* = 203 of 206 total samples). In all but three collection zones, each sample was a unique MLG. The moderate diversity observed within these collection sites appears to contradict much of the current research concerning rare or endangered plant species (Ellstrand and Elam, 1993; Gitzendanner and Soltis, 2000; Willi et al., 2006).

As populations of endemic and/or fragmented species become smaller and their distribution range limited, they often result in lower levels of genetic diversity as they undergo genetic drift, inbreeding, and limited gene flow (Ellstrand and Elam, 1993; Ouborg et al., 2010). This can, in turn, create potentially deleterious effects on the ability of these smaller populations to regenerate, which further elevates the risk of extinction. However, reduced effective population size may impact species diversity and their genetic structure in different ways depending on the biology, ecology, and genetics (Cuartas-Hernández and Núñez-Farfán, 2006). Our genetic diversity results reported here are similar to previously published study on *H. verticillatus* by Mandel (2010). However, the author reported the evidence of high genetic diversity (He = 0.48), whereas we approached it more conservatively and described our findings as moderate genetic diversity (He = 0.50), which is similar to other endemic species as reported by Nybom (2004).

Genetic diversity of the rare and endangered sunflower *H. niveus* spp. *tephrodes* (Algodones sunflower), which is native to Southern California and Mexico, was investigated by Mandel et al. (2013). The authors reported lower levels of genetic diversity (H_exp_ = 0.31) in *H. niveus* spp. *tephrodes* when compared to other rare (*H*. *verticillatus;* H_exp_ = 0.48) and endemic species [*H. porter* (Porter’s sunflower); H_exp_ = 0.62] (Table 5; Gevaert et al., 2013; Mandel et al., 2013). *Helianthus niveus* spp. *tephrodes* showed a similar genetic structure to our results in which clustering was correlated with geography, but the sampling sites were considerably further apart than in our study (Mandel et al., 2013). However, Gevaert et al. (2013) focused on *H. porteri*, which had a larger number of individuals (*n* = 200) than studies on *H. niveus* spp. *tephrodes* (*n* = 119) or *H. verticillatus* (*n* = 71) studies, which could result in biasing the outcomes of genetic diversity analyses (Table 5). Because of declining habitat (rocky outcroppings in the southeast United States) of *H. porteri*, efforts have been made regarding reintroduction of this species and estimation of spatial distribution (Gevaert et al., 2013). Although their findings indicated high genetic diversity, they found a lack of population structure.

**TABLE 5:** Comparison of sample size, genetic diversity, and genetic structure from multiple species of *Helianthus* in past studies with data generated in this study on *H. verticillatus.*

Species/status	Sample size (N)	Number of loci tested	Genetic diversity (H_exp_)	Genetic structure (*F*st)
*H. angustifolius* (common)^A^	48	11	0.34	0.17
*H. annuus* (common)^A^	12	11	0.58	N/A
*H. grosseseratus* (common)^A^	56	11	0.44	N/A
*H. niveus* spp. *tephrodes* (rare)^A^	119	11	0.31	0.17
*H. porter* (endemic)^A^	200	18	0.62	0.12
*H. verticillatus* (rare)^A^	71	11	0.48	0.12
*H. verticillatus* (rare)^B^	206 (203^C^)	14	0.50	0.24

*^*A*^Data taken from Gevaert et al. (2013) and primers from Pashley et al. (2006). ^*B*^Data generated in this study. ^*C*^Number of samples after clone correction (current study).*

The genetic diversity of *H. niveus* spp. *tephrodes* and *H. porteri* were compared with the findings for *H. verticillatus* in Mandel’s 2010 study (Mandel, 2010). In this study, similar levels of genetic diversity were calculated as reported by Mandel (2010), although the study included fewer individuals from a much larger geographic distribution (*n* = 71; across AL, GA, and TN sites). The lower number of *H. verticillatus* samples could bias the outcome of the study, therefore resulting in misrepresentation of species diversity and overall fitness (Nei and Roychoudhury, 1974; Nei, 1978). Nei and Roychoudhury (1974) speculated that lack of appropriate sample numbers can be compensated by increasing the number of loci tested, especially concerning heterozygosity and genetic distance per locus. This could also play a part in the drastic differences in species diversity as the number of loci tested in these studies varied from 11 to 18 (Table 5; Nei and Roychoudhury, 1974; Nei, 1978; Mandel, 2010; Gevaert et al., 2013; Mandel et al., 2013).

Habitat fragmentation and small population size can increase inbreeding among individuals, thus limiting gene flow, which can reduce genetic variation and population fitness (Young et al., 1996; Storfer, 1999; Willi et al., 2006). The presumed high levels of vegetative propagation in *H. verticillatus* could provide an explanation for reduced diversity within these populations (Ellstrand and Roose, 1987; Ellis et al., 2006; Mandel, 2010). However, the moderate diversity found in our study may be explained by the possibility that sexual reproduction was more frequent within these sampling sites in the past. Furthermore, the apparent diversity may be a relic of previous sexual reproduction (Esselman et al., 1999) or sexual reproduction is currently occurring but resulting in few viable seeds (Ellis, 2008).

Moderate to high genetic diversity has been found in other endangered plant systems similar to that of *H. verticillatus* (Widén and Andersson, 1993; Ellis et al., 2006; Mandel, 2010). Studies involving *Senecio integrifolius*, an endangered species found in Sweden, showed that spatial population structure may play a large role in genetic diversity (Ellstrand and Elam, 1993; Widén and Andersson, 1993). The target species in this study exhibited high levels of diversity despite the samples coming from small fragmented populations (Widén and Andersson, 1993). The authors proposed that small populations shelter genetic diversity and when habitat destruction causes population fragmentation, many generations are required to limit variations within the populations (Widén and Andersson, 1993). *Helianthus verticillatus*, which is endemic to a limited number of sampling sites and a possible relic of a larger population, may contribute to the moderate levels of diversity observed in our study (Ellstrand and Elam, 1993; Ellis et al., 2006). The high levels of spatial and genetic structure found in our sampling sites could also be contributing to the moderate level of diversity, which can decrease linearly in organisms with a clonal mode of reproduction (Balloux et al., 2003; De Meeûs and Balloux, 2004; Halkett et al., 2005).

Linkage disequilibrium has become a very important tool in determining whether a species is reproducing asexually (clonally) versus sexually, which can influence demography and genetics of natural populations (Flint-Garcia et al., 2003; De Meeûs and Balloux, 2004; Halkett et al., 2005; Slatkin, 2008). Linkage disequilibrium is the non-random association of alleles occurring in at least two loci and can be used to understand mutations in populations as well as the effects of natural selection. Although the use of linkage disequilibrium in the past two decades has greatly increased in the studies of plants, it was more commonly reported in human-related studies (Flint-Garcia et al., 2003; Slatkin, 2008). Significant linkage disequilibrium for all but one collection zone indicated that *H. verticillatus* is more likely clonally reproduced, supporting our hypothesis of predominately vegetative reproduction (Tian et al., 2015; Kamvar et al., 2017). Evidence of a recent bottleneck reinforces the presence of high linkage disequilibrium and is caused by genetic drift and the low number of allelic combinations passed to future generations (Flint-Garcia et al., 2003). Sugarcane (*Saccharum* spp.) is an example of high linkage disequilibrium in a plant species which, like *H. verticillatus*, has been subject to a genetic bottleneck and is propagated mainly through asexual means (Flint-Garcia et al., 2003).

In accordance with our hypothesis, genetic structure was based upon the sampling locations. However, there was some admixture observed between the two sites, with the majority present in collection zone R1. One plausible explanation is that the R1 collection zone is located on the forested barrier between site two (R1–9) and site one (C1–5). With the close proximity (less than 1 km) of these two sites, it is possible that pollinators in the forested site could be transferring pollen between the two locations. It would be presumed that native and honey bees would be the primary pollinators for this as well as other sunflowers, but very little research has been done on that subject in wild *Helianthus* species (Heiser et al., 1969). Some bee species travel only to neighboring plants and have a flight distance of no more than 3 m (Schmitt, 1980). However, physical barriers and geographic distance between populations could prevent pollinators moving among individual populations (Schmitt, 1980; Loveless and Hamrick, 1984).

Habitat fragmentation almost always leads to a reduction in population numbers, which inevitably leads to a reduction in diversity and a population bottleneck (Amos and Harwood, 1998). There was significant heterozygote excesses in our population when analyzed by DAPC and STRUCTURE as well as by sampling location, which suggested a population bottleneck caused by a reduction in individuals (Barrett and Kohn, 1991). Because STRUCTURE and BAPS analyses assume that the loci studied are in Hardy-Weinberg equilibrium (HWE), the evidence of heterozygote excesses could contradict those results (Pritchard et al., 2000; Hubisz et al., 2009). However, when analyzed by parameters set by DAPC and Bruvo’s distance, neither of which assume the presence of HWE, the results pointed to two genetic clusters in accordance with STRUCTURE (Bruvo et al., 2004; Corander et al., 2008; Jombart et al., 2010). The moderate levels of diversity (H_exp_ = 0.50) in these collection zones suggested that the bottleneck occurred recently and a reduction in variation has not been able to take place (Barrett and Kohn, 1991). Likewise, clonal reproduction may have led to an increase in heterozygosity by way of somatic mutations over time, similar to the apomictic fern *Dryopteris remota* (wood fern) or tree species like *Populus tremuloides* (trembling aspen) (Antolin and Strobeck, 1985; Judson and Normark, 1996; Schneller et al., 1998; Ally et al., 2008). Because of this reduction in the number of individuals and the spatial structure of these populations, they are at an increased risk for inbreeding depression and a loss of overall fitness (Amos and Balmford, 2001).

### Conservation Implications and Future Research

There is a lack of understanding of the biology of *H. verticillatus* and its relation to the environment. Further research into seed viability and *in vitro* propagation could prove to be crucial for preservation of this species in seed banks and botanic gardens (O’Donnell and Sharrock, 2017; Volis, 2017). The continued monitoring of these populations will provide critical data of the differences of fitness from site to site and how this species interacts with its environment. With this and previous research, coupled with an expanded understanding of the ecology and predicament of *H. verticillatus*, and potentially investigating paternity analyses, a comprehensive recovery plan is expected to promote this rare plant’s persistence in its native habitat (USFWS, 2002; Bowen, 2011).

Combining previous research on *H. verticillatus*, other *Helianthus* species, and data on endangered and recovered plants, we recommend the following five actions: (1) continued research of the biology and ecology of *H. verticillatus*; (2) placement of this species in botanical gardens and seed banks; (3) exploration for unknown populations; (4) continued monitoring of known populations; and (5) *in vitro* propagation and germplasm preservation.

## Data Availability Statement

The datasets generated for this study can be found in the Dryad – https://doi.org/10.5061/dryad.f4qrfj6s5.

## Author Contributions

RT, PW, and DH conceived and planned the experiments. TE carried out the experiments, took the lead in writing the manuscript and other authors provided critical feedback, edits, and helped shape the research, analysis and manuscript. TE, CW, and DH planned and carried out the simulations. TE, RT, CW, PW, and DH contributed to sample collection and preparation. All authors contributed to the interpretation of the results.

## Conflict of Interest

The authors declare that the research was conducted in the absence of any commercial or financial relationships that could be construed as a potential conflict of interest.

## Supplementary Material

FIGURE S1 Diagram of sampling (grid) employed at sites one (upper) and two (lower). Site one consisted of five contiguous 1 × 1 m plots. At site two, a 3 × 3 m grid was created. The circles represent sampling locations within the grid. Sample names (RX.X) are inlayed within each circle and collection zones (R1–9) are differentiated by color. Empty circles represent locales where no plant was present and no sample was taken (lower diagram).

FIGURE S2 Population structure and clustering for *Helianthus verticillatus* by collection zone using the Bayesian analysis program BAPS. The first graph (upper) is assuming two clusters and the second graph (lower) is assuming three clusters.

TABLE S1 Pairwise population matrix of Nei’s unbiased genetic distance of *Helianthus verticillatus* samples from two sampling sites (C and R) split into 14 collection zones.

TABLE S2 Pairwise population differentiation for *Helianthus verticillatus* samples from two sampling sites (C and R) divided into 14 subpopulations using 14 microsatellite loci. *F*st values are based on 9,999 permutations.
