# Supplementary figures and images for: Genetic Diversity and Conservation Status of Helianthus verticillatus, an Endangered Sunflower of the Southern United States
Source: Front Genet. 2020 May 15;11:410. doi: 10.3389/fgene.2020.00410 (PMC7243268; doi:10.3389/fgene.2020.00410)

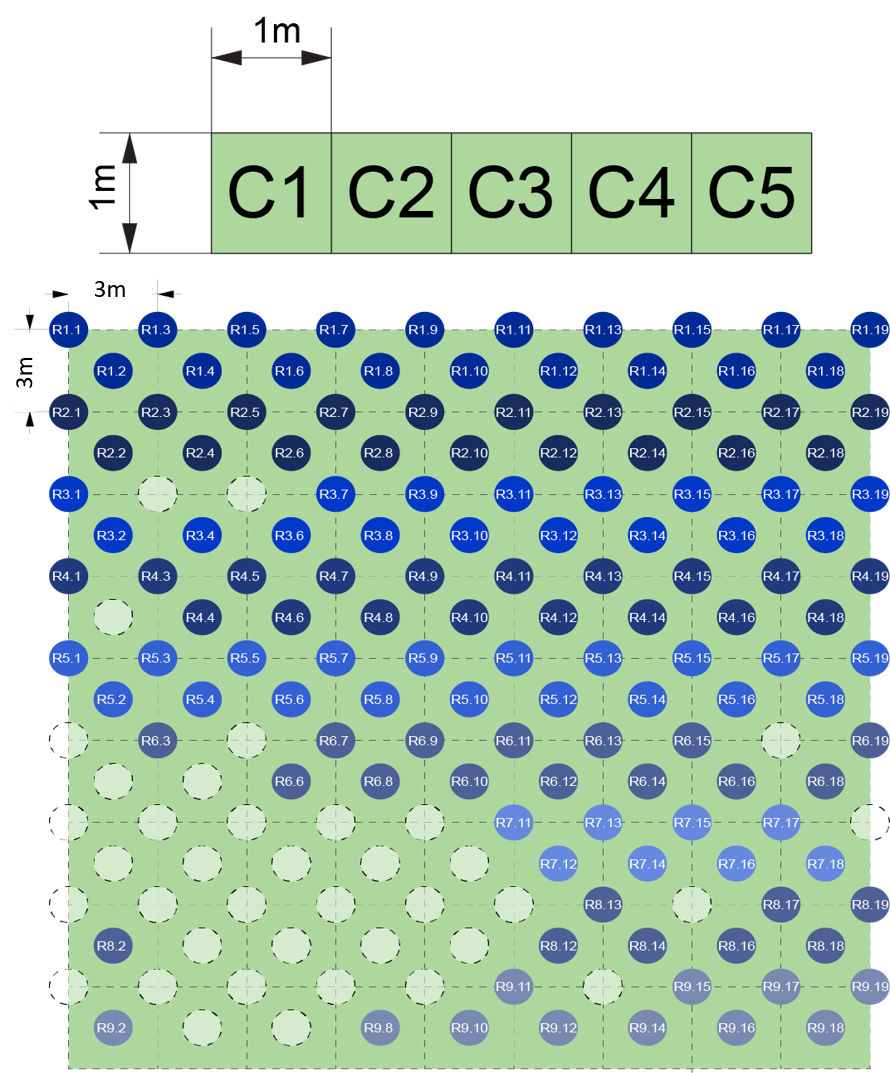

Supplement: FIGURE S1 — Diagram of sampling (grid) employed at sites one (upper) and two (lower). Site one consisted of five contiguous 1 × 1 m plots. At site two, a 3 × 3 m grid was created. The circles represent sampling locations within the grid. Sample names (RX.X) are inlayed within each circle and collection zones (R1–9) are differentiated by color. Empty circles represent locales where no plant was present and no sample was taken (lower diagram). [file Image_1.TIF]

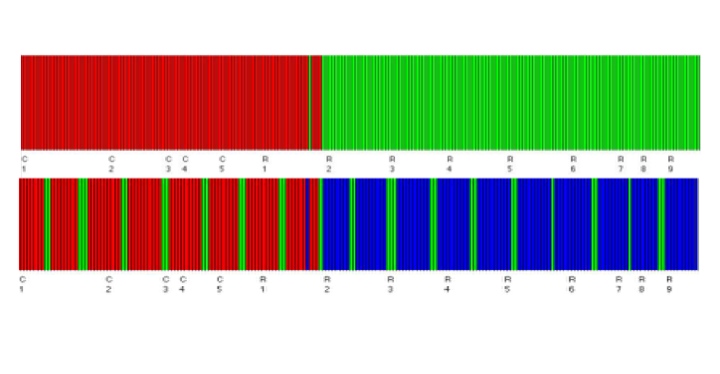

Supplement: FIGURE S2 — Population structure and clustering for Helianthus verticillatus by collection zone using the Bayesian analysis program BAPS. The first graph (upper) is assuming two clusters and the second graph (lower) is assuming three clusters. [file Image_2.TIFF]
