# Supplementary material for: Genetic Diversity and Conservation Status of Helianthus verticillatus, an Endangered Sunflower of the Southern United States
Source: Front Genet. 2020 May 15;11:410. doi: 10.3389/fgene.2020.00410 (PMC7243268; doi:10.3389/fgene.2020.00410)
Supplement: TABLE S1 — Pairwise population matrix of Nei’s unbiased genetic distance of Helianthus verticillatus samples from two sampling sites (C and R) split into 14 collection zones. [file Data_Sheet_1.docx]

| **Table S1.** Pairwise population matrix of Nei's unbiased genetic distance of *Helianthus verticillatus* samples from two sampling sites (C and R) split into 14 collection zones. | | | | | | | | | | | | | |
| --- | --- | --- | --- | --- | --- | --- | --- | --- | --- | --- | --- | --- | --- |
|  |  |  |  |  |  |  |  |  |  |  |  |  |  |
|  | **C1** | **C2** | **C3** | **C4** | **C5** | **R1** | **R2** | **R3** | **R4** | **R5** | **R6** | **R7** | **R8** |
| **C2** | 0.14 | − |  |  |  |  |  |  |  |  |  |  |  |
| **C3** | 0.25 | 0.12 | − |  |  |  |  |  |  |  |  |  |  |
| **C4** | 0.33 | 0.18 | 0.15 | − |  |  |  |  |  |  |  |  |  |
| **C5** | 0.32 | 0.18 | 0.17 | 0.02 | − |  |  |  |  |  |  |  |  |
| **R1** | 0.26 | 0.22 | 0.17 | 0.19 | 0.18 | − |  |  |  |  |  |  |  |
| **R2** | 0.66 | 0.66 | 0.60 | 0.50 | 0.57 | 0.33 | − |  |  |  |  |  |  |
| **R3** | 0.56 | 0.56 | 0.53 | 0.43 | 0.48 | 0.35 | 0.14 | − |  |  |  |  |  |
| **R4** | 0.66 | 0.59 | 0.53 | 0.45 | 0.51 | 0.38 | 0.14 | 0.10 | − |  |  |  |  |
| **R5** | 0.60 | 0.56 | 0.51 | 0.39 | 0.44 | 0.30 | 0.09 | 0.09 | 0.06 | − |  |  |  |
| **R6** | 0.64 | 0.62 | 0.58 | 0.45 | 0.51 | 0.35 | 0.08 | 0.14 | 0.11 | 0.08 | − |  |  |
| **R7** | 0.52 | 0.53 | 0.61 | 0.62 | 0.68 | 0.44 | 0.18 | 0.22 | 0.23 | 0.23 | 0.17 | − |  |
| **R8** | 0.89 | 1.06 | 0.84 | 0.92 | 0.99 | 0.68 | 0.42 | 0.38 | 0.46 | 0.44 | 0.42 | 0.37 | − |
| **R9** | 0.93 | 0.92 | 0.86 | 0.95 | 1.03 | 0.91 | 0.73 | 0.70 | 0.70 | 0.77 | 0.69 | 0.52 | 0.27 |

| **Table S2.** Pairwise population differentiation for *Helianthus verticillatus* samples from two sampling sites (C and R) divided into 14 subpopulations using 14 microsatellite loci. *F*st values are based on 9,999 permutations. | | | | | | | | | | | | | |
| --- | --- | --- | --- | --- | --- | --- | --- | --- | --- | --- | --- | --- | --- |
|  |  |  |  |  |  |  |  |  |  |  |  |  |  |
|  | **C1** | **C2** | **C3** | **C4** | **C5** | **R1** | **R2** | **R3** | **R4** | **R5** | **R6** | **R7** | **R8** |
| **C2** | 0.09 | − |  |  |  |  |  |  |  |  |  |  |  |
| **C3** | 0.08 | 0.05 | − |  |  |  |  |  |  |  |  |  |  |
| **C4** | 0.12 | 0.06 | 0.05 | − |  |  |  |  |  |  |  |  |  |
| **C5** | 0.16 | 0.09 | 0.08 | 0.02 | − |  |  |  |  |  |  |  |  |
| **R1** | 0.11 | 0.10 | 0.05 | 0.07 | 0.09 | − |  |  |  |  |  |  |  |
| **R2** | 0.21 | 0.21 | 0.12 | 0.18 | 0.22 | 0.12 | − |  |  |  |  |  |  |
| **R3** | 0.17 | 0.18 | 0.12 | 0.17 | 0.21 | 0.12 | 0.07 | − |  |  |  |  |  |
| **R4** | 0.21 | 0.20 | 0.12 | 0.17 | 0.21 | 0.13 | 0.07 | 0.06 | − |  |  |  |  |
| **R5** | 0.19 | 0.19 | 0.11 | 0.15 | 0.19 | 0.11 | 0.06 | 0.05 | 0.04 | − |  |  |  |
| **R6** | 0.22 | 0.25 | 0.17 | 0.23 | 0.27 | 0.15 | 0.08 | 0.08 | 0.08 | 0.06 | − |  |  |
| **R7** | 0.15 | 0.19 | 0.18 | 0.23 | 0.26 | 0.13 | 0.09 | 0.08 | 0.09 | 0.08 | 0.06 | − |  |
| **R8** | 0.19 | 0.25 | 0.19 | 0.25 | 0.28 | 0.16 | 0.13 | 0.10 | 0.13 | 0.11 | 0.13 | 0.10 | − |
| **R9** | 0.23 | 0.28 | 0.21 | 0.28 | 0.31 | 0.21 | 0.20 | 0.18 | 0.19 | 0.19 | 0.20 | 0.15 | 0.09 |
